# Serum sodium level fluctuations following the resection of childhood‐onset craniopharyngioma

**DOI:** 10.1002/brb3.3430

**Published:** 2024-03-03

**Authors:** Yuqi Miao, Kaiyu Fan, Xiaojiao Peng, Si Li, Jiahui Chen, Yu Wei, Yaxian Deng, Chengsong Zhao, Qingfeng Wu, Ming Ge, Jian Gong, Di Wu

**Affiliations:** ^1^ Department of Endocrinology, Genetics and Metabolism Beijing Children's Hospital, Capital Medical University, National Center for Children's Health Beijing China; ^2^ Department of Pediatric Neurosurgery Beijing Tiantan Hospital, Capital Medical University Beijing China; ^3^ Department of Neurosurgery Beijing Children's Hospital, Capital Medical University, National Center for Children's Health Beijing China; ^4^ State Key Laboratory of Molecular Development Biology Institute of Genetics and Developmental Biology, Chinese Academy of Sciences Beijing China; ^5^ Department of Pediatric Beijing Tiantan Hospital, Capital Medical University Beijing China; ^6^ Beijing Children's Hospital, Capital Medical University, National Center for Children's Health Beijing China; ^7^ Beijing Key Laboratory for Genetics of Birth Defects Beijing China

**Keywords:** craniopharyngioma, hypernatremia, hyponatremia, serum sodium level, triphase response

## Abstract

**Background:**

Craniopharyngiomas are low‐grade malignancies (WHO I) in the sellar region. Most cases of childhood‐onset craniopharyngioma are adamantinomatous craniopharyngioma, and neurosurgery is the treatment of choice. Affected patients have postoperative complications, including water and electrolyte disturbances, because these malignancies develop near the hypothalamus and pituitary gland. Determining postoperative serum sodium fluctuation patterns in these patients can reduce postoperative mortality and improve prognosis.

**Objective:**

To measure changes in serum sodium levels in pediatric patients who underwent craniopharyngioma surgery and identify influencing factors.

**Methods:**

This retrospective study measured the serum sodium levels of 202 patients aged 0–18 years who underwent craniopharyngioma resection in Beijing Tiantan Hospital and Beijing Children's Hospital and identified predictors of severe hyponatremia and hypernatremia.

**Results:**

The mean age of the cohort was 8.35 ± 4.35 years. The prevalence of hypernatremia, hyponatremia, and their severe forms (serum Na^+^ > 150 mmol/L and serum Na^+^ < 130 mmol/L) within 14 days after surgery was 66.3%, 72.8%, 37.1%, and 40.6%, respectively. The mean postoperative serum sodium level showed a triphasic pattern, characterized by two peaks separated by a nadir. Sodium levels peaked on days 2 (143.6 ± 7.6 mmol/L) and 14 (143.2 ± 6.7 mmol/L) and reached their lowest on day 6 (135.5 ± 7.5 mmol/L). A total of 31 (15.3%) patients met the diagnostic threshold for hyponatremia and hypernatremia of the triphase response, whereas 116 (57.4%) patients presented this pattern, regardless of met the diagnostic criteria or not. The prevalence of severe hyponatremia varied depending on preoperative endocrine hormone deficiency, tumor status (primary or recurrent), and surgical approach.

**Conclusions:**

Serum sodium levels after craniopharyngioma resection in children showed a triphasic pattern in most cases. The risk of postoperative hyponatremia varied depending on preoperative endocrine hormone deficiency, tumor status (primary or recurrent), and surgical approach.

## Graphical abstract

Serum sodium levels after craniopharyngioma resection showed a triphasic pattern in most cases. The risk of postoperative hyponatremia varied depending on preoperative endocrine hormone deficiency, tumor status (primary or recurrent), and surgical approach.

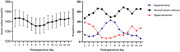

## 1 BACKGROUND

Craniopharyngiomas are low‐grade malignancies (WHO I) in the sellar region (Müller et al., 2019). Most cases of childhood‐onset craniopharyngioma are adamantinomatous craniopharyngioma (ACP), which usually involve cystic components and calcifications with or without solid components (Johnson et al., 1986). In contrast, papillary craniopharyngioma occurs predominantly in adult patients and has distinct pathological features (Muller, 2014). Craniopharyngioma primarily affects the sellar and parasellar regions adjacent to the pituitary gland, hypothalamus, optic chiasm, and third ventricle. For this reason, both craniopharyngioma itself and treatment process may impair the function of these tissues.

The initial manifestations in pediatric patients at diagnosis are usually non‐specific, including headache and/or vomiting (due to increased intracranial pressure), and visual impairment. In addition, some patients present characteristic symptoms, such as hypothalamic‐pituitary dysfunction (Muller, 2008). Surgical resection is the preferred treatment for childhood‐onset craniopharyngioma in China, including transcranial excision and transsphenoidal endoscopic endonasal surgery. In cases where total tumor resection is unsuccessful, adjuvant radiotherapy can be used to reduce recurrence (Muller, 2010).

Water and electrolyte disturbances include transient or permanent central diabetes insipidus, syndrome of inappropriate secretion of antidiuretic hormone (SIADH), cerebral salt‐wasting syndrome (CSW), and triphasic pattern (hypernatremia followed by hyponatremia and reemerging hypernatremia). Postoperative serum sodium levels fluctuate more sharply in pediatric patients, and the prevalence of a triphasic pattern, hyponatremia, and permanent diabetes insipidus is also higher, causing troubles in postoperative patients management (Pratheesh et al., 2013). Little is known about short‐term postoperative electrolyte disturbances in Chinese pediatric patients with craniopharyngioma. This study measured serum sodium levels in pediatric patients who underwent craniopharyngioma surgery in two medical centers from 2018 to 2019 and identified risk factors.

## 2 METHODS

### 2.1 Data collection

This study collected retrospective data on pediatric patients under 18 who underwent neurosurgery in the sellar region at Beijing Children's Hospital and Beijing Tiantan Hospital between January 2018 and December 2019. Age, gender, clinical manifestations at onset, surgical approach, and postoperative pathological results were analyzed. Patients with missing hospitalization records were excluded from the study. Histology confirmed all the tumors to be craniopharyngiomas.

Laboratory tests assessed pituitary function before and 14 days after surgery. Serum sodium levels were measured at 7 a.m. from postoperative days 1–14 or until the day of discharge if the hospital stay was less than 14 days. The study was approved by the Medical Ethics Committee of Beijing Children's Hospital (2020‐k‐188).

### 2.2 Definitions

Hypernatremia was defined as serum Na^+^ > 145 mmol/L and hyponatremia as serum Na^+ ^< 135 mmol/L. Severe hypernatremia was defined as serum Na^+^ > 150 mmol/L and severe hyponatremia as serum Na^+^ < 130 mmol/L. Diabetes insipidus was defined as urine volume > 3 L/m^2^/day with increased plasma osmolality (>300 mOsm/kg), hypernatremia, decreased urinary osmolality (<300 mOsm/kg), or decreased urine‐specific gravity (<1.010). The triphasic pattern was defined as postoperative diabetes insipidus, followed by hyponatremia and the recurrence of diabetes insipidus soon afterward.

### 2.3 Statistical analysis

Statistical analysis was performed using SPSS Statistics version 23.0 (IBM). Data were expressed as means and standard deviations for normally distributed continuous variables and as medians and interquartile ranges for non‐normally distributed continuous variables. Differences among groups were compared using Pearson's chi‐square test or Fisher's exact test. *p*‐Values less than .05 were considered statistically significant. The influencing factors of severe hyponatremia were assessed by multivariate logistic regression analysis.

## 3 RESULTS

### 3.1 Patient information

**FIGURE 1:**
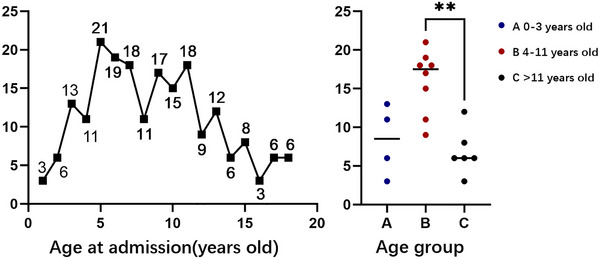
Left: age at admission. Right: distribution by age group. Group A: infants and children (0–3 years), Group B: preschoolers/schoolers (4–11 years), and Group C: adolescents (>11 years).

A total of 202 pediatric patients (130 [64.4%] males and 72 [35.6%] females) with craniopharyngioma were included in the study (shown in Figure 1 left). The mean age at disease onset was 8.35 ± 4.35 years. Patients were divided into three age groups: 0–3, 4–11, and > 11 years old. Patient distribution by age group is shown in Figure 1 right. The number of 4–11‐year old is significantly higher than the number of adolescents (*p* = .0093). Primary tumors in the sellar region were found in 163 (80.7%) patients, whereas 39 (19.3%) patients had recurrent tumors.

### 3.2 Clinical manifestations and treatment

Clinical manifestations at disease onset are shown in Table 1. The most common symptoms were headache (92, 45.5%), visual impairment (81, 40.1%), and nausea (67, 33.2%). In addition, some participants had complaints of endocrine dysfunctions, including short stature (27, 13.4%) and central diabetes insipidus (30, 14.9%). Some patients had no chief complaints and accidentally discovered the tumor during cranial imaging of trauma or other reasons. Most patients (185, 91.6%) underwent craniotomy for tumor resection, whereas the others (17, 8.4%) underwent transsphenoidal endoscopic endonasal surgery. The main pathological type was ACP (192, 95.1%). Two patients died perioperatively due to multiple organ failure.

**TABLE 1:** Clinical manifestations of disease onset.

Clinical manifestations	*n*	%
Headache	92	45.5
Nausea	67	33.2
Visual impairment	81	40.1
Convulsion	8	4.0
Central diabetes insipidus	30	14.9
Short stature	27	13.4
Accidental image finding without manifestations (e.g., brain trauma)	9	4.5
Total	202	

### 3.3 Postoperative changes in serum sodium levels and influencing factors

**FIGURE 2:**
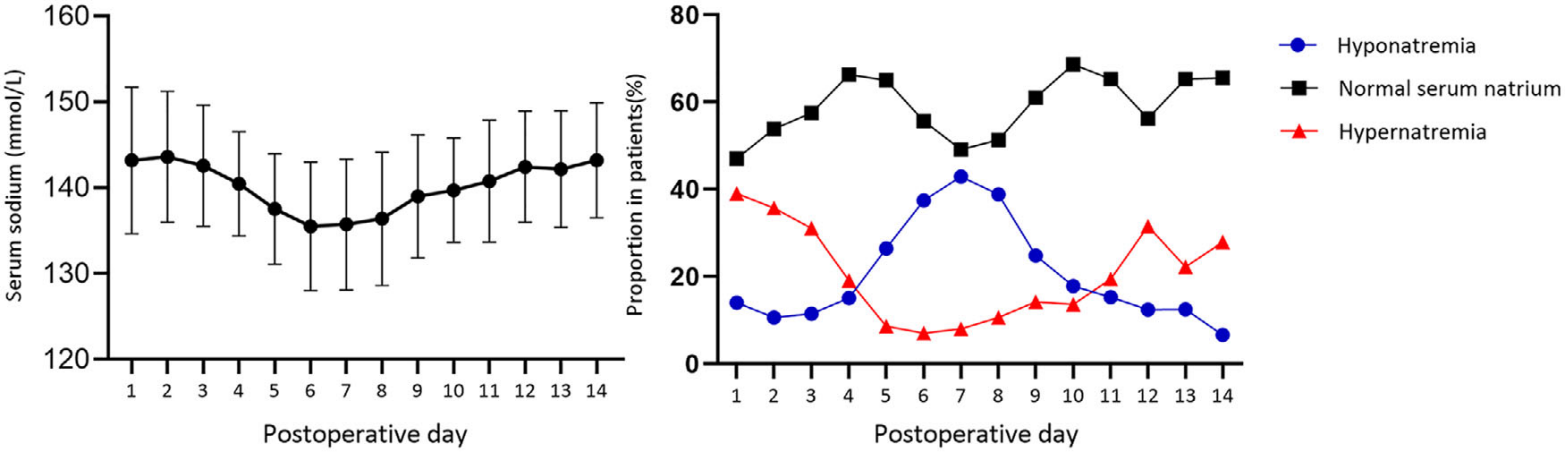
Changes in serum sodium levels in pediatric patients who underwent craniopharyngioma surgery. Left: mean sodium levels over time. Right: prevalence of hypernatremia and hyponatremia.

The fluctuations in serum sodium levels from postoperative days 1 to 14 are shown in Figure 2. The average change in sodium levels showed a triphasic pattern (two peaks separated by a nadir) (shown in Figure 2, left). Sodium levels peaked on days 2 (143.6 ± 7.6 mmol/L) and 14 (143.2 ± 6.7 mmol/L) and reached its lowest on day 6 (135.5 ± 7.5 mmol/L). In total, 116 (57.4%) patients presented this pattern, but only 31 (15.3%) reached the threshold. Hypernatremia, hyponatremia, and their severe forms (hyper and hypo) occurred in 134 (66.3%), 147 (72.8%), 75 (37.1%), and 82 (40.6%) patients, respectively. The median onset time of hypernatremia and hyponatremia was 1 (1‐2) and 4 (2‐6) days, respectively. The mean duration was 3.20 ± 2.41 for hypernatremia and 3.12 ± 1.99 for hyponatremia. Hypernatremia and hyponatremia had a bimodal pattern, the former peaked on day 1 (39.0%) and day 12(31.5%), whereas the latter peaked on day 7 (42.9%) (shown in Figure 2, right).

We evaluated numerous influencing factors for postoperative severe hyponatremia and hypernatremia, including age, preoperative pituitary hormone deficiency, tumor status (primary or recurrent), and surgical approach. The prevalence of severe hyponatremia varied depending on preoperative hormone deficiency, tumor status, and surgical approach (Table 2). The prevalence of severe hyponatremia was significantly higher in patients without hormone deficiency than in those with hormone deficiency (44.90% vs. 29.10%, *p* = .042). In addition, the prevalence of severe hyponatremia was significantly lower in patients who underwent transsphenoidal endoscopic endonasal surgery than in those who underwent craniotomy (43.20% vs. 11.80%, *p* = .011). Craniotomy, the absence of preoperative hormone deficiency, and primary tumors were incorporated into the binary logistic regression model, with only the first two possessing a significant impact (Table 3).

**TABLE 2:** Influencing factors analysis of severe hyponatremia and severe hypernatremia.

Influencing factors	Group	Severe hyponatremia (%)		Severe hypernatremia (%)	
Age	0–3	40.90	*χ* ^2 ^= 2.498	66.60	*χ* ^2 ^= 1.315 *p* = .518
	4–11	43.80	*p* = .287	23.50	
	>11	30.20		3.30	
Preoperative pituitary hormone deficiency	Yes	29.10	*χ* ^2^= 4.147	34.50	*χ* ^2^= .216
	No	44.90	*p* = .042^*^	38.10	*p* = .642
Tumor status	Primary	44.40	*χ* ^2^= 5.029	37.00	*χ* ^2^= .003
	Recurrent	25.00	*p* = .025^*^	37.50	*p* = .957
Surgical approach	Craniotomy	43.20	*χ* ^2^= 6.397	36.80	*χ* ^2^= .130
	Transsphenoidal endoscopic endonasal surgery	11.80	*p* = .011^*^	41.20	*p* = .718

^*^ *p *< 0.050.

**TABLE 3:** Multivariate binary logistic regression analysis of influencing factors with severe hyponatremia.

Influencing factors	OR	95% CI	*p* Value
Preoperative pituitary hormone deficiency	2.031	1.027–4.020	.042^*^
Primary tumor	2.095	.938–4.679	.071
Craniotomy	5.52	1.198–25.431	.028^*^

^*^ *p *< 0.050.

## 4 DISCUSSION

After craniopharyngioma surgery, patients may have severe electrolyte disorders, including temporary or permanent diabetes insipidus and hypernatremia. This large retrospective study is the first to measure serum sodium levels in Chinese pediatric patients who underwent craniopharyngioma surgery and to evaluate influencing factors. Further, this study provides the theoretical basis for postoperative fluid management in patients with craniopharyngioma.

### 4.1 Triphasic pattern

Some patients had a triphasic pattern of serum sodium levels after craniopharyngioma surgery, characterized by two peaks separated by a nadir. The first peak occurred 24–48 h after surgery. Surgical intervention of connections between cell bodies and nerve terminals of ADH‐secreting neurons on the pituitary stalk, or reduced blood supply can temporarily impair ADH secreting function. Hyponatremia lasted 2–14 days, with the uncontrolled release of ADH from neuronal terminals, resulting in reduced urine output and hyponatremia. Central diabetes insipidus reappeared approximately 14 days after resection, associated with the degeneration of ADH‐releasing neurons and decreased ADH storage in the posterior pituitary. This phase is often permanent (Loh & Verbalis, 2007).The prevalence of the triphasic pattern in pediatric patients with craniopharyngioma is 23%–29%, which is usually higher than that in adult patients (Muller, 2010).

Although the prevalence of this pattern was lower in our study than that of previous reports, approximately 50% of our patients showed this pattern, regardless of prior clinically diagnosed hypernatremia or hyponatremia. Sodium levels were usually higher on days 1–4 and 11–14 after surgery, during which hypernatremia occurred much more often. The median time of hyponatremia onset was postoperative day 4 in most cases and lasted approximately 3 days, consistent with a previous study (Finken et al., 2011). This result indicates that several patients had this pattern but did not meet the diagnostic criteria for hyponatremia and hypernatremia, underscoring the need for timely transfer fluid therapy to restore water and electrolyte balance in these patients.

### 4.2 Hypernatremia

Postoperative hypernatremia is common in craniopharyngioma patients, and the prevalence of postoperative temporary diabetes insipidus is 80%–100% in these patients (Caldarelli et al., 2005; Grill et al., 2014; Poretti et al., 2004). The etiology of transient diabetes insipidus is complex. Preoperative tumor masses cause damage to the pituitary gland and surgical traumatic neuronal damage often occurs. The limited blood supply to the hypothalamic–pituitary region can impair neuronal function and decrease the secretion of ADH, resulting in diabetes insipidus and hypernatremia. In addition, inappropriate fluid therapy and impaired thirst regulation after surgery can cause short‐term hypernatremia (Crowley et al., 2007).

The prevalence of postoperative hypernatremia in our cohort was 66.3%, lower than previous reports. This discrepancy may be related to adequate fluid therapy and oral treatment with desmopressin (1‐deamino‐8‐d‐arginine vasopressin [DDAVP]). Although all patients received treatment, most had diabetes insipidus, and hypernatremia control was difficult. Long lasting hypernatremia and high proportion of patients suffering from severe postoperative hypernatremia demonstrate the difficulty in managing diabetes insipidus. Thus, clinicians should measure fluid intake, urine output, urine osmotic pressure, and serum sodium levels every 4–6 h until clinical improvement is observed and should promptly perform oral replacement therapy with DDAVP to reduce urine volume.

In addition to temporary diabetes insipidus after surgery, 63%–76% of patients develop permanent diabetes insipidus after craniopharyngioma surgery (Finken et al., 2011; Hussein et al., 2021). This condition is related to degeneration and functional loss of ADH‐releasing neurons in the posterior pituitary lobe due to surgical injury. Such patients usually have anterior pituitary hormone secretion disorders and require long‐term replacement of various hormones, including ADH (Andereggen et al., 2018; Capatina et al., 2018). It is necessary to adjust the DDAVP dose and fluid intake according to the patient's clinical situation and carry out long‐term, individualized management.

### 4.3 Hyponatremia

Up to 46% of pediatric patients may develop hyponatremia within 7 days after craniopharyngioma surgery (Capatina et al., 2018), and some cases of severe hyponatremia are difficult to control (Pratheesh et al., 2013). The causes of hyponatremia are complex, including SIADH, CSW, central adrenal insufficiency, hypothyroidism, excessive DDAVP dosage, and inadequate fluid therapy. SIADH is an abnormal increase in ADH secretion due to posterior pituitary neuron damage, decreasing urine volume, serum sodium, and blood osmotic pressure, and increasing urinary sodium, urine osmotic pressure, and blood volume. Patients with CSW have increased urinary sodium, polyuria, and potentially hypovolemic hyponatremia. The prevalence of CSW after craniopharyngioma surgery is up to 2.9% (Grill et al., 2014; Jameel et al., 2021), whereas the prevalence of CSW in pediatric patients with brain tumors is up to 5% (Grill et al., 2014; Williams et al., 2016).

Treatments for SIADH and CSW vary depending on blood volume status. In SIADH, fluid intake should be limited, and urine output is expected to increase. Symptomatic severe hyponatremia can be managed using 3% NaCl and arginine vasopressin receptor antagonists (vaptans). Tolvaptan is a selective inhibitor of vasopressin receptor 2, which reduce water reabsorption and increase urine excretion. It can control symptoms of severe refractory hyponatremia that cannot be alleviated by hypertonic saline infusions (Tuli et al., 2017). It is safe to correct hyponatremia by 6–8 mmol/L per day. However, the rapid correction of hyponatremia can be life‐threatening and leads to osmotic demyelination syndrome (Gürbüz et al., 2019). For patients with CSW, immediate fluid resuscitation is recommended to restore blood volume and increase serum Na^+^ levels. Fluid therapy with 0.9% or 3% NaCl is appropriate in most cases. In addition, fludrocortisone increases serum sodium and controls polyuria (Papadimitriou et al., 2007).

Before DDAVP treatment in patients with transient diabetes insipidus, therapies for adrenocortical deficiency and hypothyroidism can be administered to decrease the risk of hyponatremia. Fluid intake and medication regimens should be adjusted according to urine output during the whole procedure in order to avoid hyponatremia or cerebral edema caused by the violent fluctuation of serum sodium level (Edate & Albanese, 2015).

### 4.4 Influencing factors of abnormal water and sodium homeostasis

The prevalence of severe hyponatremia was higher in patients without preoperative pituitary hormone deficiency and patients with primary tumors. The reason may be that these patients have normal preoperative hypothalamus‐pituitary function, which enhanced ADH releasing ability after surgery. Patients who undergo the transcranial approach for tumor resection usually have larger tumors with stronger adhesion to surrounding tissues, increasing surgical difficulty and damage to hypothalamic‐pituitary tissues. Thus, a larger proportion of neurons may be affected and cause the higher prevalence of severe hyponatremia. These characteristics highlight the need to monitor fluid intake and output, serum sodium levels, and serum or urine osmotic pressure in patients with primary tumors, patients without preoperative pituitary hormone deficiencies, and patients undergoing craniotomy.

## 5 LIMITATIONS

As a retrospective study, urine osmotic pressure and volume were excluded from the analysis for the incomplete record in clinical practice. In addition, the treatment plans, such as fluid formula and oral treatment with DDAVP or glucocorticoids, tend to be individualized according to the patient's needs due to high variability in serum sodium levels across patients. Thus, it is difficult to perform measurements and analyses on an individual basis.

## 6 CONCLUSIONS

This study measured the variability in serum sodium levels in children who underwent craniopharyngioma resection. Postoperative changes in serum sodium levels presented as a triphasic pattern, characterized by two peaks separated by a nadir. Most patients developed hypernatremia or hyponatremia within 14 days after surgery. Hypernatremia showed a bimodal pattern, and hyponatremia peaked approximately 7 days after surgery. The prevalence of severe hyponatremia in our cohort varied depending on preoperative hormone deficiency, tumor status (primary or recurrent), and surgical approach. Although surgical techniques in removing craniopharyngioma have been improved, postsurgical fluid and electrolyte disturbances are common difficulties in perioperative management. The increased understanding of fluctuation pattern may reduce the risk of life‐threatening perioperative complications and be helpful to improving long‐term prognosis.

## AUTHOR CONTRIBUTIONS

The study idea and design were conceived by Di Wu. Yuqi Miao, Kaiyu Fan, Xiaojiao Peng, Si Li, Yu Wei, and Yaxian Deng collected clinical data included in this study. Yuqi Miao and Jiahui Chen analyzed the data and produced the figures and tables. All authors participated in the writing of the paper and critical discussions, read, and approved the final manuscript.

## CONFLICT OF INTEREST STATEMENT

The authors have no conflicts of interest to declare.

## FUNDING INFORMATION

The work was supported by Beijing Municipal Science&Technology Commission(Z210010) and National Nature Science Foundation of China(62276027).

### PEER REVIEW

The peer review history for this article is available at https://publons.com/publon/10.1002/brb3.3430.

## Data Availability

The datasets for this article are not publicly available due to concerns regarding participant/patient anonymity. Requests to access the datasets should be directed to the corresponding authors.
